# The Address in Medicine

**Published:** 1901-08-03

**Authors:** 


### THE ADDRESS IN MEDICINE.

On Wednesday the Address in Medicine was delivered by J. F. Goodhart, M.D., F.R.C.P.

Likening the living body to the clay between the potter (the doctor) on the one hand, and the wheel (the blind guidance of animal life) on the other, he showed that this plastic clay, this living body, has its own peculiarities, each life being individual and distinct. There are probably in the whole universe, he said, no two living beings exact in counterpart. If we could see into the minute details of the working of the machine, not only do no two men think alike, but no two men have hearts alike, no two men have a liver that pours out its bile exactly alike. And yet all our parts are one. So absolutely one that it is impossible to suffer bad pain in two separate parts of the body at the same time—one comes and the other goes. So absolutely one that when you think hard your feet grow cold, so one that when one member of the body suffers all the members suffer with it; so one that the temporary failure to lock or unlock the points at the many junctions and sidings will disarrange the whole.

But to understand the wonder of this "clay" one must remember that behind all, or rather before it, is this curious unfathomable mystery "the boon of life." "I needs must live," the body seems to say. Some cynic asks where is the necessity. But that is life, or all we know about it. The "needs must" live, an unconscious blind unquenchable energy that carries on the being irrespective of his will, and which, blending with his conscious will, renders the clay at once so plastic, and yet too at one and the same time so unimpressionable. Many and many a mistake does the doctor make by under-estimating the vitality of life. This irrepressible living energy will carry on to old age a very weakly flesh. Think of the disappointed ones in the race, those who are tired and full of pain from early years even to old age. There are many of these who must be very weary of life, but the motive force of their being will not let them go. Now take these two points, the intricacy of the mechanism of the animal body and the indomitable animal spirit that innervates it. How do these react upon the practice of medicine?

It is clear, without labouring the point, that a body so composite as ours is so very delicate a machine that there must be many and many a case presented to us where we do not, many even where we cannot, know what is the matter. Our difficulties then become appalling when we attempt to treat diseases affecting such a frame. There has been much interesting, and perhaps interested, talk of late about water-tube boilers, their intricacy of working, and their excessive wear and tear, and the difficulty of their repair without stopping the ship. Now supposing that such a machine as our modern warship could be worked automatically with hatches down, and that the only positive knowledge to be obtained of the condition of its entrails was by the observation and analysis of the smoke, and the bilge water, by post-mortem examination at the breaking-up of old vessels and the recording where each had gone to pieces, would the progress of knowledge, think you, be very rapid in the evolution of the science and art of ship building? Would the feeling of power to deal with the defects of the machinery as they arose be very robust in any engineer who had had much experience?

What is our position in regard to that real and intimate knowledge of the action of the liver, for example, which we require to enable us to treat its diseases? We still give our blue pill and podophyllin, and so on, and speak with early innocence of "touching" the liver, and so still perpetuate the idea that certain remedies go for the liver and increase its various secretions. I don't say this is all wrong, or that the remedies are not valuable in the conditions for which they are given, but all the positive experimental evidence that we possess I think goes to show that these remedies are chiefly intestinal in their action.

Take next the vital element and its bearing upon treatment. This is one side of what may be called the individuality of the patient. When Mrs. Smith asked her doctor why it was that a particular pain possessed her, he is said to have replied with ready wit and no less truth, "Madam, it is because you are Mrs. Smith." And the lady no doubt thought her doctor a very amusing man, but she had not a glimmer of the great truth that had been administered in such an excellent coating. But, indeed, that "because you are Mrs. Smith" constitutes one of, if not the most insuperable of the difficulties to framing any system of precise medicine; and as long as the world lasts, this variability of the living force, this individuality, will prevent the attainment of the popular desire—a cut-and-dried remedy.

In discussing the relations between doctor and patient, Dr. Goodhart said that in the daily treatment of disease, with all the manifold details of the aches and pains that flesh is said to be heir to, one cannot but feel that there is room for improvement both on the side of the patient and on that of the doctor. It may be that the duration of life is now longer than it was, but medicine is in no less request, nor is an ailing public less in evidence than before. On the contrary, we are growing more sensitive about our health and more impatient, and more than ever do we demand a remedy even when, as in some cases of phthisis, the greater part of an organ is destroyed and restoration of its health is impossible.

The sick man now always wants to know too much. He wants to know what is the matter with him when it is not possible to tell him; moreover, he will have an answer, and if not he thinks the doctor an ignoramus, and calls in someone else. Oh if men would only think a little more. Any fool can give a name to a disease, if, as is too often the case, the letter satisfieth. But what if the name is wrong, and the name determines an important line of treatment; what if the letter killeth? The public will have the disease ticketed, even when there is no means of identification; and having got a name for his complaint the patient thinks the physic tumbles out of the same slot, and that between the two he will be cured offhand. There is no idea of doubt—none that the powers of medicine are limited in all sorts of ways, and that waiting for developments or subsidences is the only skilful course. Waiting is described as "nothing is being done for me," and someone else is called in with a "Can't you do something?"

The morbid sensitiveness of people in the present day is well shown by the rapidity with which they fly to medicine. The number of new drugs that are daily launched upon us is bewildering in the extreme. Then, too, with what impatience do men and women in the present day rush into the not always sufficiently repellent arms of surgery! A little pain unnerves them, and all they know of surgery is its successful side. It is a day of great things, and why should they not have the benefit of these advances? And so with an ache here or a pain there they undergo an operation. The energy of life that I have spoken of knows nothing of risks; knows nothing of shock; will hear nothing of waiting and rest in bed, and the disappointment in consequence is often considerable.

And now for the potter who has more or less to fashion this clay as it rolls along under the momentum of the wheel of life. What are we, the doctors, doing in this impatient, restless age to stem the tide, to stay the panic, to bid the people keep its head? I do not doubt that every one of us does his best for the man that consults him, but I am not sure that in attending to the exigencies of the immediate present we do sufficiently take heed of the future; and first among our failings may be put a morbid readiness to detect disease. Engaged as we are in this pursuit there comes a risk that we too little appreciate the wide range of health; that is, how good a state of health is compatible with numberless slight and even sometimes considerable departures from normal. I will take an illustration from the heart. Over and over again in the present day a heart is said to be strained, or weak, or dilated, or even diseased as to its valves from a want of sound appreciation of what is to be considered health, not for the general, but for the particular. One would almost think from all the talk one hears about dilatation of the heart, strains in healthy young people from trivial causes, the grave conclusions that are based upon, perhaps, some slight displacement of the impulse, etc., that the heart is so fragile an organ that it needs to be coddled from the cradle to the grave. I hate the term weak heart. It coffins, or, worse, throws useless upon society, many an otherwise useful life.

Again, take the stomach with its "catarrhs" and its "dilatations." Their best treatment often is to let them severely alone. But the public won't have it. Who does not know the difficulty there is in preventing people from undergoing a serious operation for the purpose of stitching these harmless mobilities—for it is only quite exceptional that it is otherwise—into their places? It is the same in many another region: throats and noses suffer terribly from this lust of operation that has beset the public. Ears are now being swept into the panic, and I incline to think that the only region of our art that preserves its proper decorum is that of ophthalmic surgery, and it, I believe, reaps the reward of well-doing that is usual in this topsy-turvy world in being regarded by the *élite* as somewhat old-fashioned, and so it is supposed to be the thing to go abroad to skim the cream of skill. Coming, then, to drugs, why do we give them? To cure disease, you answer at once, and think the question unnecessary. But wait a minute; drugs are given for several other reasons, some of which are far less free from criticism. Sometimes because the patient will not be happy till he gets them; sometimes to hide our ignorance, or to mark time while we watch and wait; and then we often give drugs as an experiment in the hope that they may do good. All treatment by drugs is more or less of an experiment. That we cannot help. So long as one man differs from another it must be so. Many an ailment badly needs a remedy, and who knows but what in each new drug some human ill may find alleviation? What I would discountenance is the giving drugs by rule of thumb.

As an instance of routine treatment consider the case of gout. The open-air treatment of consumption, of which we are hearing much at the present day, is also bidding fair to come under the baneful influence of routine. What, think you, does the consumptive and his friend see in this? He sees a residence for a few months in a home, and a cure at the end of it. Is that what he has any chance of obtaining? Certainly not. And in proportion to the exaggerated hope will come the bitterness of the disappointment to the sick, and the discredit to us. The benefit to be obtained in these sanatoria is that there will be learned a habit of life; what we mean by plenty of good food and plenty of fresh air; and having learned his lesson the tuberculous man will need to practise it all the rest of his life. There is no cure in this treatment as the sick man understands cure; for although it is true that there is no disease that is more often arrested than phthisis, it is equally true that there is no disease that has a more inveterate tendency to relapse, and I very much fear, when you come to strike the balance between arrest and relapse, that the latter has the best of it. Therefore if the open-air treatment is to take its real place and be of any abiding value, the principles of the sanatorium must be introduced into the home. Other examples of routine are the giving of intestinal antiseptics, and of bromides in epilepsy.

Dr. Goodhart ended his most eloquent and interesting address by urging that we should not belittle the value of our advice by allowing the public to exalt so greatly the supremacy of physic.

